# A class of human exons with predicted distant branch points revealed by analysis of AG dinucleotide exclusion zones

**DOI:** 10.1186/gb-2006-7-1-r1

**Published:** 2006-01-13

**Authors:** Clare Gooding, Francis Clark, Matthew C Wollerton, Sushma-Nagaraja Grellscheid, Harriet Groom, Christopher WJ Smith

**Affiliations:** 1 Department of Biochemistry, University of Cambridge, 80 Tennis Court Road, Cambridge CB2 1GA, UK; 2 Advanced Computational Modelling Centre, and ARC Centre for Bioinformatics, University of Queensland, Australia

## Abstract

**Background:**

The three consensus elements at the 3' end of human introns - the branch point sequence, the polypyrimidine tract, and the 3' splice site AG dinucleotide - are usually closely spaced within the final 40 nucleotides of the intron. However, the branch point sequence and polypyrimidine tract of a few known alternatively spliced exons lie up to 400 nucleotides upstream of the 3' splice site. The extended regions between the distant branch points (dBPs) and their 3' splice site are marked by the absence of other AG dinucleotides. In many cases alternative splicing regulatory elements are located within this region.

**Results:**

We have applied a simple algorithm, based on AG dinucleotide exclusion zones (AGEZ), to a large data set of verified human exons. We found a substantial number of exons with large AGEZs, which represent candidate dBP exons. We verified the importance of the predicted dBPs for splicing of some of these exons. This group of exons exhibits a higher than average prevalence of observed alternative splicing, and many of the exons are in genes with some human disease association.

**Conclusion:**

The group of identified probable dBP exons are interesting first because they are likely to be alternatively spliced. Second, they are expected to be vulnerable to mutations within the entire extended AGEZ. Disruption of splicing of such exons, for example by mutations that lead to insertion of a new AG dinucleotide between the dBP and 3' splice site, could be readily understood even though the causative mutation might be remote from the conventional locations of splice site sequences.

## Abstract 2

Exons with predicted branch points were identified from a large dataset of human exons and the importance of these branch points for splicing was verified.

## Background

Pre-mRNA splicing is an essential step in eukaryotic gene expression as well as an important regulatory point via the process of alternative splicing [1-4]. Removal of introns and splicing together of exons is essential for the generation of functional mRNAs from pre-mRNAs. The importance of splicing is attested to by the observation that at least 15% of human genetic diseases are caused by mutations within the consensus sequence elements at the exon-intron boundaries, which are important for specifying the splice sites [5-7]. The 5' splice site consists of a nine-nucleotide consensus containing the invariant GU dinucleotide at the start of the intron. At the 3' end of the intron, usually within about 40 nucleotides upstream of the exon, there are three elements (in 5' to 3' order): a branch point sequence (BPS); a polypyrimidine tract (PPT); and the 3' splice site itself, which consists of the invariant AG dinucleotide at the end of the intron, usually preceded by a pyrimidine residue. Recognition of these consensus elements by various *trans*-acting protein and RNA splicing factors leads to assembly of the spliceosome, within which the two chemical steps of splicing occur [8]. In the first step the 2'-OH group of the branch point adenosine attacks the 5' splice site, leading to formation of the 5' exon and the intron lariat intermediates. In the second step, the 3'-OH of the 5' exon attacks the 3' splice site, leading to production of the spliced RNA and the excised intron, still in the lariat configuration.

Although the consensus splice site elements are essential, they are degenerate in many positions, and have insufficient information content to specify correctly the ends of long metazoan introns [8]. This deficit is partly addressed by the presence of auxiliary splicing enhancer sequences, commonly found within exons (exonic splicing enhancers), which activate splicing of adjacent splice sites [9,10]. A number of RNA binding (for example [11]) and functional SELEX (selective evolution of ligands by exponential enrichment) experiments [12-14], as well as computational analyses [15,16], have been used to identify various classes of exonic splicing enhancers (see Matlin and coworkers [3] for a discussion).

**Figure 1:**
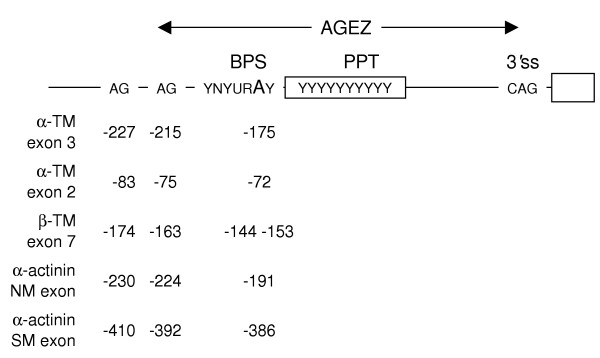
Sequence arrangement at dBP exons. The locations of several dBPs that have been mapped *in vitro *are shown, along with the locations of the first and second AG dinucleotides upstream of the 3'ss. In experimentally verified cases of dBP exons the BPS and PPT can be located hundreds of nucleotides upstream of the 3'ss. Because step 2 of splicing in these introns involves a scanning process from the BPS to locate the 3'ss at the first downstream AG, the region between the 3'ss and the BPS is devoid of AG dinucleotides. Upstream of the BPS, AGs appear no longer to be excluded, as indicated by the locations of second AGs upstream of the 3'ss. Here we refer to the region between the 3'ss and the first upstream AG as the AG exclusion zone (AGEZ). BPS, branch point sequence; dBP, distant branch point; PPT, polypyrimidine tract; 3'ss, 3' splice site.

The conventional arrangement of elements within 40 nucleotides at the 3' ends of introns is not obligatory. A number of alternatively spliced exons have been characterized in which the BPS has been mapped 100-400 nucleotides from the 3' splice site [17-21] (Figure 1), and artificial splicing substrates have also been created with this arrangement [22]. In some cases, these distant branch points (dBPs) are close enough to the upstream exon to promote mutually exclusive splicing [19,20]. In all cases that have been investigated, regulatory elements have been found to lie between the dBP and the exon [20,21,23-26]. These introns can be characterized as 'AG independent' in the sense that step 1 of splicing occurs without the need for the 3' splice site AG [22]. The 3' splice site is then located during step 2 of splicing by a linear search for the first AG dinucleotide downstream of the dBP [27-29]. Consequently, a hallmark of experimentally verified dBP exons is an extended region immediately upstream that is devoid of AG dinucleotides. We refer to this region as the 'AG exclusion zone' (AGEZ). In these verified cases the BPS and PPT are located toward the 5' end of the AGEZ, and upstream of the AGEZ AG dinucleotides appear to occur at a normal frequency (Figure 1). Exceptions to the simple BPS to AG scanning model can occur when AG dinucleotides occur relatively close (<12-15 nucleotides) to the BPS and these can be bypassed, or when the 3' splice site has two or more closely spaced (<12 nucleotides) AGs, in which case the preceding nucleotide plays an important role in their competition [28,30].

We devised a simple algorithm that can be used to locate putative dBPs. First, we define the AGEZ upstream of each exon by conducting a 3' to 5' search from the 3' splice site for the first upstream AG. In the small number of cases in which AG dinucleotides exist before -12, we ignore them and continue the search for the first AG beyond -12. We then search for probable candidate BPs in a region defined by the AGEZ but also including a further approximate 15 nucleotides upstream. This additional 15 nucleotides is also considered because AGs very close to the BPS can be bypassed by the spliceosome during step 2 of splicing [28,30]. Candidate dBPs are identified by consensus sequence (see Materials and methods, below) and by the presence of an adjacent PPT, and are often close to the 5' end of the AGEZ. Here, we have applied this approach globally by classifying human exons according to the size of their AGEZ. We find that there is an excess of exons with large AGEZ, and that putative dBP exons exhibit a higher than average prevalence of alternative splicing.

## Results

### Analyzing introns for AG exclusion zones

**Figure 2:**
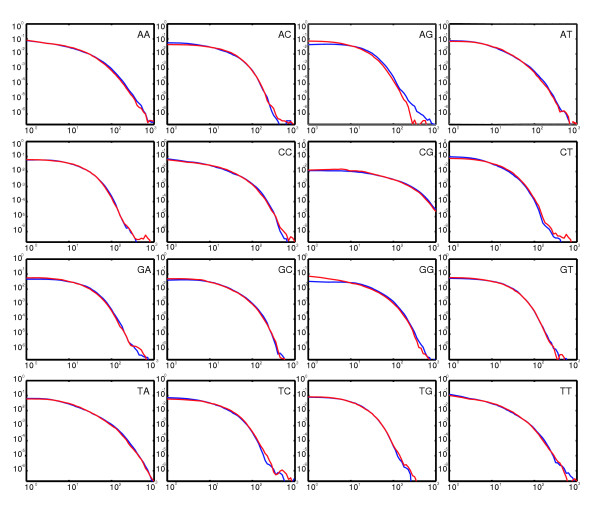
Distribution of dinucleotide exclusion zones. Shown is the distribution of dinucleotide exclusion zones (mod-EZ) upstream of 49,876 human exons (having excluded cases in which the intron was less that 350 nucleotides). Y-axis: log [number of exons]. X-axis: log [size of mod-EZ]. Data are normalized to give a probability density function, which gives the probability that an exon chosen at random will have an exclusion zone of a given size; the area under each curve is 1. Blue lines: first exclusion zone (mod-EZ_1_), measured from -25 (relative to the 3' splice site) to the first upstream occurrence of the particular dinucleotide (see Materials and methods). Red lines: second exclusion zone (mod-EZ_2_), measured from -25 relative to the end of the mod-EZ_1_. AG shows the largest variance between mod-EZ_1 _and mod-EZ_2_. Data was sorted into bins of logarithmically increasing widths rendered discrete (bin width 10 at ~100; bin width 100 at ~1,000), with final bin counts divided by bin width and by the total number of exons, followed by application of a three-point averaging filter to produce the given plots. See Materials and methods for full details.

We analyzed a set of 67,334 human exons from AltExtron (version 3; based on GenBank release 147) [31,32] for the size of dinucleotide exclusion zones upstream of their 3' splice site. When plotted as log(number of exons) versus log(size of EZ), the distribution of AGEZ values did not obviously exhibit a simple excess of high values compared with the curves for the other dinucleotides. However, frequencies of dinucleotide occurrence can be affected by many factors other than splicing. Notably, the general scarcity of CpG dinucleotides leads to very large CGEZs upstream of many exons (Figure 2). We therefore compared the distributions of 'first' and 'second' exclusion zones upstream of exons (EZ_1 _and EZ_2_, respectively). In the experimentally verified dBP exons, the distance between first and second AGs upstream of the 3' splice site is much shorter than between the 3' splice site and the first upstream AG (Figure 1). Because scanning for the 3' splice site takes place downstream from dBPs, we expect a selective pressure against AG dinucleotides between dBPs and the 3' splice site, and conversely a general lack of selective pressure against AGs upstream of BPs. On this basis we expect the AGEZ_1 _distribution to be biased toward higher values when compared with AGEZ_2 _distributions. Although our method avoids potential problems that can arise due to heterogeneity in base composition dynamics between the gene sequences (by having one EZ_1 _datum and a corresponding EZ_2 _datum derived from each intron sequence, for each dinucleotide, under consideration), it remains a concern that heterogeneity in base composition within an intron could affect our analysis. The common location of the PPT immediately upstream of the 3' splice site is an obvious candidate for introducing this sort of bias. In order to control for this to some extent and for other methodological reasons (see Materials and methods, below), we present these distribution comparisons (Figure 2) using modified definitions of the EZ_1 _and EZ_2 _(mod-EZ_1 _and mod-EZ_2_), and having restricted the dataset to exclude introns of less than 350 nucleotides in length (see Materials and methods, below). Briefly, mod-EZ_1 _is the distance from -25 (relative to the 3' splice site) to the first upstream occurrence of a particular dinucleotide. A further upstream shift of 25 nucleotides from the 5' end of the mod-EZ_1 _is then carried out before commencing the search to define mod-EZ_2_.

Comparison of the mod-EZ_1 _and mod-EZ_2 _profiles revealed the curves for each dinucleotide to be (visually) very similar in all cases except for AG (Figure 2; compare blue and red lines). For the AG dinucleotides there was a readily identifiable shoulder on the mod-EZ_1 _distribution at higher values (≥ 100 nucleotides) compared with the mod-EZ_2 _distribution. There are 279 mod-AGEZ_1 _exons at 100 nucleotides or greater compared with 148 for the mod-AGEZ_2 _curve, giving a χ^2 ^value of 116 (*P *≈ 0). This confirms the visual impression that the mod-AGEZ_1 _and mod-AGEZ_2 _distributions are significantly different. The excess of exons with large AGEZ_1 _represents a group of potential dBP exons. We note that some other dinucleotides also exhibit lesser but still statistically significant differences under equivalent analysis (in particular bias toward TC and CT in the mod-EZ_1 _region; further details may be found under Materials and methods, below). As an initial test of whether the excess of the exons with mod-AGEZ_1 _≥ 100 are associated with dBPs, we repeated the analysis of mod-AGEZ_1 _distributions having first split the intron data-set into two groups according to whether or not they had an AG dinucleotide between -12 and -25 with respect to the 3' splice site. The expectation is that exons with an AG between -12 and -25 (the 'plus' group) cannot have a dBP (otherwise the additional AG would be used as the 3' splice site). Consistent with this expectation, the percentage of mod-AGEZ_1 _values ≥ 100 nucleotides was 0.68% for introns without an AG in the -12 to -25 region (the minus group), but only 0.23% for those with an AG. With a null hypothesis that the minus group should generate the same statistics as the plus group, we observe the null hypothesis to be false, with a χ^2 ^of 356 (*P* ≈ 0).

In the data set proper there are 838 exons with AGEZ ≥ 100. We estimate that between one-half and one-fifth of these indicate dBPs (see details under Materials and methods, below); with 838 of 67,334 introns having an AGEZ_1 _≥ 100, we expect that approximately 1/160 to 1/400 introns have dBPs. Taking an 'average' human gene to have eight introns, this reduces to between 1/20 and 1/50 genes having at least one dBP (as defined here).

To facilitate manual examination of large AGEZ exons, we restrict consideration to those exons with AGEZ ≥ 150 (165 cases). Our data are available online [33], with separate files for the starting data set, and for exons with AGEZ ≥ 150. Among the exons with AGEZ ≥ 150 were exon 11 of the human PTB gene (AGEZ = 381, IDB1087423.10917), for which we have some *in vitro *evidence for use of a dBP [21]. Likewise, the equivalent exon from the neuronal specific paralog nPTB/brPTB [34,35] was identified (AGEZ = 438, predicted branch point at -389, IDB1145220.85254). Other dBP exons from the α-tropomyosin and α-actinin genes, which were experimentally verified for the rat genes and appear to be conserved, were not in the current build of AltExtron. Many of the exons with large AGEZ (≥ 150) had a clear potential dBP located toward the upstream end of the AGEZ with no obvious candidate BPS close to the 3' splice site. For example, IDB1152764.11013 has an AGEZ of 220 with a TACTAAC sequence at -214 and an adjacent PPT. The mouse ortholog has an AGEZ of 247 and a consensus TACTAAC BPS at -214. In other cases, large AGEZs did not appear to be related to splicing, with no obvious candidate BPS/PPT toward the 5' end of the AGEZ, whereas good candidates were in the conventional location. For example, IDB1079466.8106 has an AGEZ of 369 nucleotides. However, this appears to be due to a repetitive element upstream of the 3' splice site. Because this element lacks AG dinucleotides there is a large AGEZ, and AGs further upstream are still widely spaced. The only good candidate BPS is at -17. Instructively, the mouse orthologous exon has an AGEZ of only 31 nucleotides and a predicted BPS at -24. Intermediate between these extremes are multiple examples that might have dBPs, but that will require careful experimental verification. A striking example is tyrosine phosphatase sigma (IDB1087363.1770), which has an AGEZ of 1126 (the entire intron is only 1132 nucleotides) and potential dBPs at -1079, -829 and -288. The closest potential BPS that scores above threshold is at -171. The mouse orthologous exon has an AGEZ of 229 nucleotides with a predicted dBP at -192.

### Testing predicted distant branch points

**Figure 3:**
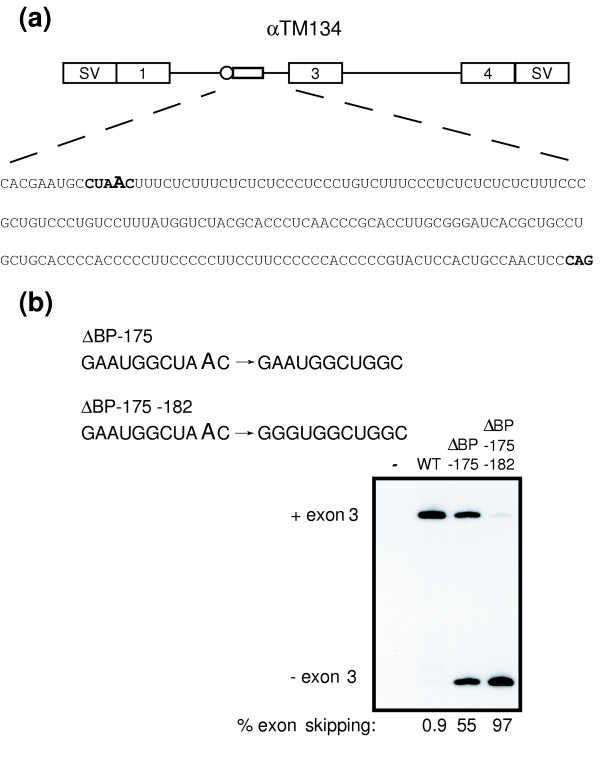
Verifying the exon trapping and mutagenesis approach for identifying distant branch points. The rat α-tropomyosin minigene (TS3St) and a derivative (ΔBP-175), in which the previously determined dBP of exon 3 had been mutated, and an additional mutant (ΔBP-175 -182) were transfected into HeLa cells. Splicing of transiently expressed RNA was analyzed by RT-PCR with a [^32^P]labeled primer in the PCR reaction. dBP, distant branch point; RT-PCR, reverse transcriptase polymerase chain reaction; WT, wild type.

Definitive mapping of branch points can be achieved by *in vitro *splicing followed by primer extension from a position downstream of the branch point; reverse transcriptase is arrested one base before the branched nucleotide [36]. However, this approach is limited to transcripts that splice efficiently *in vitro*. We therefore decided to target candidate dBPs by mutagenesis in exon trapping vectors. This approach identifies nucleotides that influence exon inclusion but does not definitively prove the branch point location. However, it has the distinct advantage of being more widely applicable. To validate the approach we first used a minigene construct containing rat α-tropomyosin exons 1, 3 and 4 (Figure 3). The dBP of exon 3 has been mapped *in vitro *to the A at 175 nucleotides upstream of exon 3, which lies within a good consensus context (ggCTAAC) [19,37]. When transfected into HeLa cells exon 3 was included to more than 99% (Figure 3b, wild type). Mutations of A to G at positions -175 and -176 led to approximately 50% exon skipping, which is consistent with mutation of the authentic dBP but suggests that use of a cryptic dBP was able to sustain the residual exon splicing (Figure 3b). Previous *in vitro *splicing with mutant transcripts had indicated that A -182 could sometimes be used as a dBP (Scadden ADJ, Smith CWJ, unpublished data). Consistent with this, mutation of the dBPs at -175 and -182 abolished exon 3 splicing. This established that mutagenesis in exon trapping vectors could be used to identify dBPs, but it also emphasized that activation of nearby cryptic dBPs might limit the magnitude of the observed effect.

**Figure 4:**
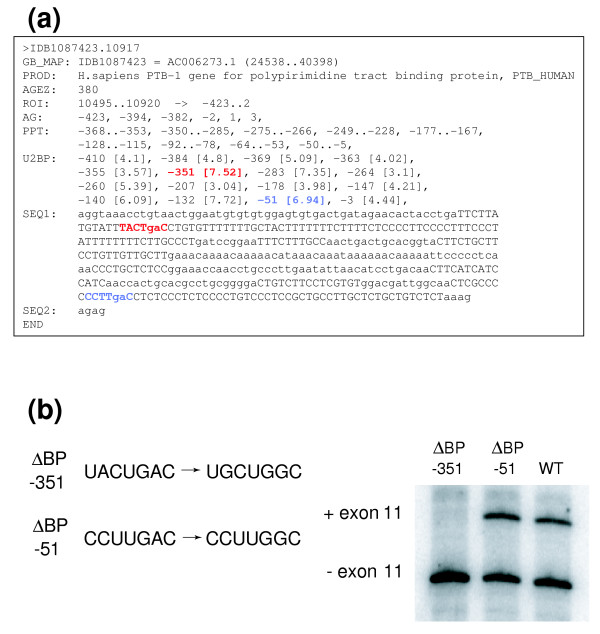
Verification of the predicted dBP of PTB exon 11. **(a) **Output for PTB exon 11 from our prototype dataset. 'AGEZ' gives the size of the AGEZ; 'AG' gives the positions of three AGs upstream of the 3' splice site and two downstream. -2 is the 3' splice site. 'PPT' and 'U2BP' give the positions of predicted PPT and BPS, with bit scores in square brackets for BPS. 'SEQ1' is the sequence from the third upstream AG to the 3' splice site, whereas 'SEQ2' is the exon sequence to the second downstream AG. Predicted PPTs are in capitals. See Materials and methods for more detailed explanation of terms. Potential BPS that were mutagenized are indicated in red and blue. **(b) **PTB exon 11 and flanking intron sequences were cloned in an EGFP exon trapping vector [21]. Mutants ΔBP-351 and -51 contained the indicated mutations in potential branch points. Constructs were transfected into HeLa cells, and RNA analyzed by reverse transcriptase polymerase chain reaction. Splicing of exon 11 was abolished in ΔBP-351. AGEZ, AG exclusion zone; BPS, branch point sequence; dBP, distant branch point; PPT, polypyrimidine tract.

Candidate dBP exons and flanking introns were cloned into EGFP (enhanced green fluorescent protein) and TM (α-tropomyosin) exon trapping vectors, and potential branch points targeted by A to G mutations. Splicing was analyzed by reverse transcriptase polymerase chain reaction (RT-PCR) after transient transfection of HeLa cells. We first tested exon 11 from the PTB gene (IDB1087423.10917). *In vitro *splicing has previously demonstrated that there is an active BPS/PPT more than 187 nucleotides upstream of the exon, but splicing of full length transcripts was too inefficient to allow BPS mapping [21]. This exon provides a challenging test for predicting dBPs. The AGEZ is 381 nucleotides in length, within which there are at least seven putative BPS (Figure 4). We predicted that the BPS is at -351 on the basis of the following: location toward the 5' end of AGEZ; the high scoring sequence UACUGAC (7.52 bits) is a perfect match to the BPS consensus heptamer, including the possibility for complete base pairing with U2 snRNA; and an adjacent uridine-rich PPT [21]. PTB exon 11 was included to a level of about 25% in an EGFP exon trapping vector (Figure 4). Mutation of the predicted -351 BPS (UACUGAC to UGCUGGC) completely abolished exon inclusion. In contrast, mutation of a potential branch point 51 nucleotides upstream of the exon (-51 CCUUGAC to CCUUGGC) had no effect, despite the fact that this is a high scoring BPS, has an adjacent polypyrimidine tract, and at -51 is only just beyond the conventional 40 nucleotides distance from the 3' splice site.

**Figure 5:**
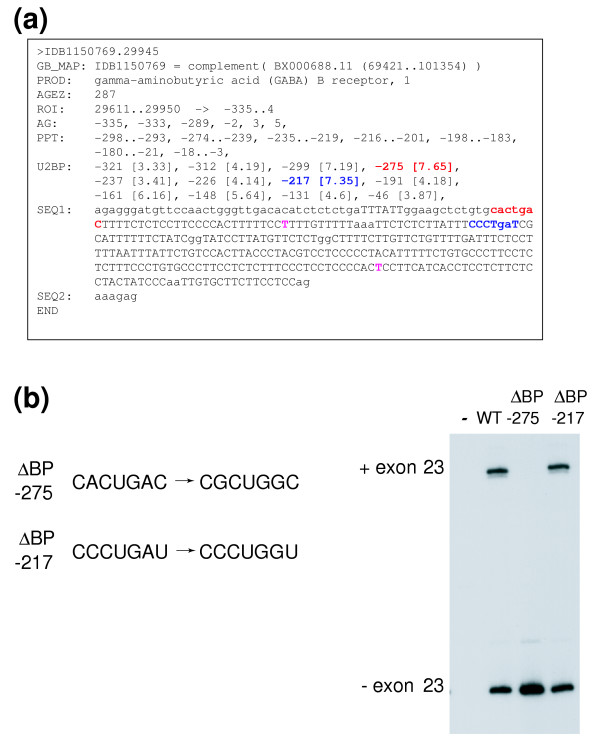
Verification of the predicted dBP of GABBR1 exon 23. **(a) **Output for GABBR1 exon 23 from our prototype data set. The various field labels are as described in the legend to Figure 4. The two magenta colored Ts are sites of single nucleotide polymorphisms and can be T or C. The potential BPS indicated in bold red and blue were mutagenized. **(b) **GABBR1 exon 23 and flanking intron sequences were cloned in the EGFP exon trapping vector [21]. Mutants ΔBP-275 and -217 contained the indicated mutations in potential branch points. Constructs were transfected into HeLa cells, and RNA analyzed by reverse transcriptase polymerase chain reaction. Splicing of exon 23 was abolished in ΔBP-275.

Next we tested two exons that had been newly identified within the group of large AGEZ exons. Exon 23 from the GBBR1 gene, which encodes the B subunit of the γ-aminobutyric acid receptor (Figure 5), has an AGEZ of 288 nucleotides. The highest scoring BPS is at -275, with an adjacent extensive PPT. This exon was inserted into both the EGFP and TM exon trapping vectors. In both vectors the exon was partially included in spliced mRNA. Exon inclusion was completely abolished by mutation of the -275 BPS (CACUGAC to CGCUGGC). In contrast, mutation of the next high scoring BPS at -217 (CCCUGAU to CCCUGGU) had no effect on exon inclusion.

**Figure 6:**
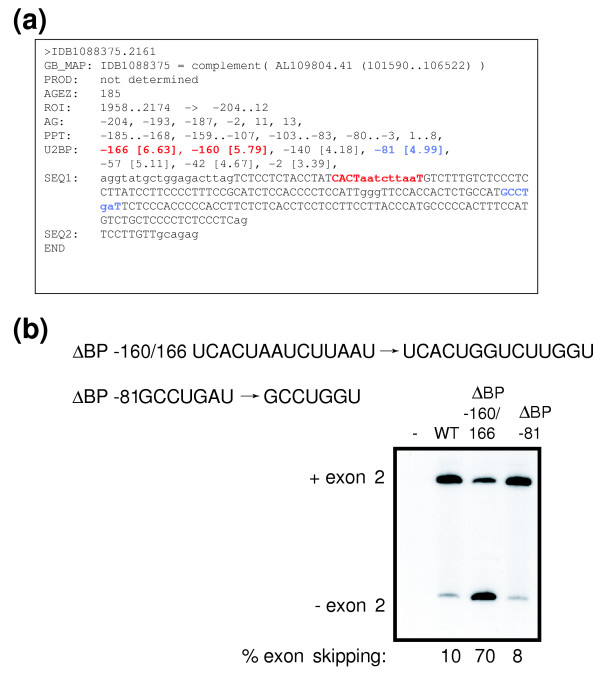
Verification of the predicted dBP of IDB1088375 exon 2. **(a) **Output from our prototype data set. The various field labels are as described in the legend to Figure 4. The potential BPS indicated in bold red and blue were mutagenized. **(b) **IDB1088375 exon 2 and flanking intron sequences were cloned in the EGFP exon trapping vector [21]. Mutants ΔBP-160/-166 and -81 contained the indicated mutations in potential branch points. Constructs were transfected into HeLa cells, and RNA analyzed by reverse transcriptase polymerase chain reaction. Splicing of exon 2 was reduced in ΔBP-160/-166, but not in -81.

Finally, we tested exon 2 of a gene encoding a novel protein (IDB1088375.2161; Figure 6). The AGEZ was 185 nucleotides, with the highest scoring potential dBPs at -160 and -166 adjacent to a PPT. We mutated the possible dBPs at -160 and -166 together (ΔBP -166/-160) and also a potential BPS at -81, which was followed by an unbroken PPT to the 3' splice site. Mutation at -81 had no effect, with about 90% exon inclusion as in the wild type. In contrast, exon inclusion was reduced to about 30% in ΔBP -166/-160. This indicates that the dBP is located at -160 and/or -166, but it also indicates that some splicing can proceed using another BPS (for example using A -170 or A -140).

### Prevalence of alternative splicing in candidate distant branch point exons

All experimental examples of dBP exons are alternatively spliced [17-21], and it is our expectation that a dBP is likely to indicate that an exon is alternatively spliced under at least some circumstances. We therefore analyzed the prevalence of observed alternative splicing (as seen in the AltExtron data set) as a function AGEZ size.

**Figure 7:**
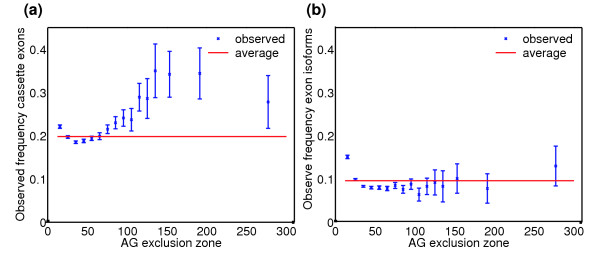
Prevalence of alternative splicing as a function of AGEZ size. For both plots, acceptor sites were excluded from consideration if there was another acceptor site ≤ 40 nucleotides upstream (see Materials and methods). In order to constrain the domain of the plots, all AGEZ values greater than 300 nucleotides were taken as 300 nucleotides. For both plots the standard error was calculated as sqrt(*r*·(*n *- *r*)/*n*), with *n *being the total number of acceptor sites/introns in the group, and *r *being the number of these seen to undergo alternative splicing of the defined type. See Materials and methods for further details. **(a) **Frequency of observed cassette exon alternative splicing as a function of the AGEZ for considered acceptor sites. The overall average is 19.8% (red line). The three data points representing AGEZ ≥ 150 nucleotides correspond to 197 exons with an average 32.5% observed cassette alternative exons. **(b) **Frequency of observed 3' splice site exon isoform alternative splicing as a function of the AGEZ for considered acceptor sites. The overall average is 9.6% (red line), with the first data point representing 8,657 exons having AGEZ values between 12 and 19 inclusive, and with 15.1% of these having observed acceptor site isoforms (intriguingly these are not a consequence of examining the downstream of two closely spaced acceptor sites because these have been excluded). AGEZ, AG exclusion zone.

First, we examined observed cassette exon type events (including mutually exclusive events) versus AGEZ (Figure 7a). Exons with AGEZ ≥ 100 nucleotides had a significantly higher frequency of observed alternative splicing, compared with the much larger number of exons with AGEZ up to 100 nucleotides (*P *= 0.002; see Materials and methods, below). The higher observed frequency of alternative splicing among large AGEZ exons is probably a conservative reflection of the alternative splicing propensity of dBP exons due to the following: all inferences of alternative splicing based upon expressed sequence tags (ESTs) are heavily restricted by the incomplete coverage and end biases of ESTs [38], and not all large AGEZ are associated with dBPs. We therefore suspect that the true prevalence of alternative splicing among dBP exons will be far higher. We also observed a higher prevalence of cassette exon events associated with very short AGEZs. The presence of two closely spaced AG dinucleotides is important for cassette skipping of exon 3 of *Drosophila sex-lethal*; if the upstream of the two AGs is mutated the the exon is constitutively included [39,40]. The group of short AGEZ cassette exons may be candidates for a similar form of regulation.

As a comparison, we observed the level of acceptor site exon modification (extension or truncation at the 3' splice site) type alternative splicing events versus AGEZ (Figure 7b). The median level was around 8% and was fairly uniform. Exons with large AGEZ did not exhibit elevated levels of this type of alternative splicing event. However, the group of exons with shortest AGEZ had a 15% observed level of alternative splicing. This spike at low AGEZ values had been considerably more pronounced prior to the following: ignoring any AGs in the last 12 nucleotides of an intron in the determination of the AGEZ; and the exclusion from the analysis (for Figure 7) of acceptor sites ≤ 40 nucleotides downstream of another acceptor site (data not shown). These filtering steps removed a large number of acceptor site isoforms involving small truncations or extensions, including the class of so-called NAGNAG splicing events [31,41] that result from competition between closely spaced AGs during step 2 of splicing [28,42]. It is noteworthy that, even after restricting the analysis in this way, there remained a modest spike at low AGEZ values. Further examination of this phenomenon is beyond the scope of this report but will be examined thoroughly in future work.

### Mutations within the AG dinucleotide exclusion zones

**Figure 8:**
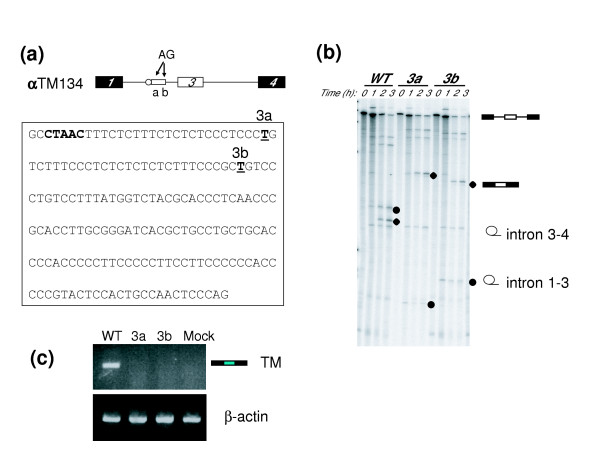
Mutations that insert AG dinucleotides in a large AGEZ impair gene expression. **(a) **Rat α-tropomyosin (TM) minigene constructs and sequence between exon 3 branch point (in bold) and 3' splice site CAG. The underlined Ts are positions where mutagenesis to A created a new AG dinucleotide in mutants 3a and b. **(b) ***In vitro *spliced [^32^P]labelled RNA was analyzed by phosphorimaging after denaturing PAGE. The fully spliced 134 product is indicated by the black diamonds, and the intron lariat resulting from excision of the intron between exons 1 and 3 by the open circles. The sizes of these two bands varied, consistent with use of the first AG downstream of the dBP for splicing of exon 3. **(c) **Reverse transcriptase polymerase chain reaction analysis of transiently expressed constructs in HeLa cells. No bands corresponding to skipping or inclusion of exon 3 using either AG dinucleotide were observed in mutants 3a and 3b. The wild type construct shows a band corresponding to spliced exons 1-3-4. WT, wild type;

There are a number of instances in which human disease is associated with mutations that introduce new AG dinucleotides a short distance upstream of the usual 3' splice site (for example [43,44]). Use of the new AG as the 3' splice site leads to insertion of one or more additional peptides, and may cause a frameshift thus potentially leading to nonsense mediated decay (NMD). Insertion of AG dinucleotides at most positions within the extended AGEZ of the rat α-TM exon 3 leads to use of the new AG as the 3' splice site *in vitro *using single intron substrates [27,28]. Exons with dBPs are therefore likely to be vulnerable to mutations within the AGEZ. To test the possible impacts of mutations that create new AG dinucleotides within a large AGEZ, we took TM minigenes containing TM exon 3 flanked by exons 1 and 4 and inserted AG dinucleotides at 149 or 121 nucleotides upstream of exon 3 (Figure 8; mutants 3a and 3b, respectively). The effect upon splicing was analyzed *in vitro *and *in vivo*. In HeLa nuclear extract we found that splicing of the mutant substrates occurred with similar efficiency to wild type, and the major splicing pathway involved use of exon 3. However, step 2 of splicing in each case used the newly inserted AG, as had been seen previously with single intron substrates *in vitro *[27,28]. When constructs were transfected into HeLa cells the levels of the product from the mutant constructs were undetectable at PCR cycle numbers used to detect wild-type product (Figure 8). With further cycles of amplification a small residual amount of spliced product could be detected in which the normal 3' splice site of exon 3 had been used (data not shown). The variation between the *in vitro *and *in vivo *data might be connected to the differences between cotranscriptional splicing *in vivo *and post-transcriptional splicing *in vitro*. However, the simplest interpretation is that splicing *in vivo *also occurs predominantly to the upstream AG, but that the products of this reaction are degraded efficiently. These model substrates illustrate that mutations throughout extended AGEZs can have catastrophic effects upon gene expression; however, analysis of *in vivo *steady state RNAs might not give any clue that disruption of gene expression is at the level of splicing.

## Discussion

Characterization of exons by upstream AGEZs provides a novel perspective for branch point prediction. This approach contrasts with conventional methods, which usually search for probable branch points within a fixed distance of the 3' splice site, sometimes using a 3' to 5' polarity for the search (for example [45]). Although the number of exons with very large AGEZ is relatively small (165 with AGEZ of 150 nucleotides or greater in our data set), there is a much larger number of exons with AGEZ of 80 nucleotides and more (2,264 cases), which is likely to include many exons with dBPs well beyond the conventional 40 nucleotides distance from the 3' splice site.

Some dBPs can be predicted by an almost mechanical application of a 5' to 3' search from the 5' end of the AGEZ (Figures 4, 5, 6). This was the case with GABBR1 exon 23, for which the AGEZ was 287 nucleotides and a high scoring dBP, subsequently verified by mutagenesis, was located at -275 (Figure 5). PTB exon 11 was slightly more complex in that the AGEZ is 380 nucleotides and, in addition to the verified dBP at -351, there were two other high scoring potential dBPs at -384 and -369, and the latter even had an adjacent PPT (Figure 4). However, the -351 BPS was higher scoring than either of the upstream candidates and its adjacent PPT is extensive and uridine rich, whereas the predicted PPT adjacent to -369 has a number of purine interruptions. The PTB, GABBR1, and IDB1088375 systems provide an attractive illustration of the applicability of the AGEZ approach to identifying dBP exons. However, many of the other large AGEZ exons do not have such readily predictable dBPs. In some cases there are multiple potential dBPs, and in others there are few or no obvious candidates.

One of our aims in future work will be to improve the computational prediction of dBPs taking into account additional information relating to the quality of the branch point and PPT sequence, and the distance separating possible branch point and PPT elements. Some of these approaches have already been adopted [45]. However, further improvements in prediction should be facilitated by the experimental verification of some of the more 'difficult' dBP exons. Another useful factor to consider is phylogenetic conservation. The BPS of human-mouse orthologous pairs have been found to be more highly conserved for alternative than constitutive exons [45]. Comparison of mouse orthologs of the human exons whose dBPs we verified here (Figures 4, 5, 6) suggests that conservation of a large AGEZ can help to focus in on a dBP even when basic local alignment search tool (BLAST) alignments do not detect significant sequence matches. For example, BLAST detected only a 24 nucleotides match immediately upstream of GBBR1 exon 23, even though the mouse had an AGEZ of 264 and predicted dBP at -225 (compared with 287 and -275 for human). Another striking example, as we previously noted [21], is the *Fugu *PTB exon 11. Its AGEZ of 590 and predicted dBP at -566 is remarkable in an organism noted for its compact genome.

We have focused on the use of AGEZs to identify unusually distant BPS. However, this approach may be a generally useful first step in prediction of all BPS. Previous BPS prediction approaches have typically used an arbitrary distance upstream of the 3' splice site within which to search for potential BPS. For example, both AltExtron [31,32] and the successful BPS procedure described by Ast and coworkers [45] restricted their searches to 100 nucleotides upstream of the exon. Defining the AGEZ as the first step in BPS prediction may help to focus the search zone to a much shorter region in many cases, in addition to the obvious advantage of locating dBPs that would otherwise be missed.

The significance of the group of probable dBP exons that we identified is twofold. First, we identified a group of exons with an increased probability of being alternatively spliced (Figure 7a). In contrast to computational identification of alternative splicing events by EST alignments [38], our approach is expected to identify some alternative splicing events for which there may be no existing experimental data. This is analogous to the use of extended regions of flanking conserved sequence as an indicator of alternative splicing [46-48]. For example, alternative splicing of PTB exon 11 was not recognized for a long time because the exon skipping event leads to NMD of the spliced product [21]. Characterization of the probable dBP arrangement gave us an early suggestion that exon 11 may indeed be a genuine alternatively spliced exon. We expect that the initial identification of some exons as having a probable dBP may provide an initial prediction of their alternative splicing, and that as more data becomes available the proportion of dBP exons known to be alternatively spliced will approach 100%.

The second significant point is that the dBP exons are expected to be vulnerable to mutations within the entire AGEZ. As we showed, mutations that introduce AG dinucleotides at multiple locations in the AGEZ can have highly disruptive effects. At a minimum, additional amino acids would be inserted. More catastrophically, the reading frame can be disrupted. Even in cases in which newly inserted sequence does not alter the reading frame of the spliced mRNA, the newly retained intron sequences can apparently lead to degradation. Interestingly, although mutant 3b (Figure 8) is predicted to lead to NMD, mutant 3a is not, and so degradation may result directly from the presence of the usually intronic sequences in the mRNA product. In addition, the regions between dBPs and their exons are often occupied by regulatory elements. Mutations that did not introduce AG dinucleotides could have more subtle effects by altering the appropriate regulation of exon selection, which can itself be a molecular cause of pathology [5,6]. Notably most of the experimentally verified cases of dBPs have regulatory elements between the 3' splice site and the dBP and PPT [20,21,23-26]. We have shown effects upon levels of exon inclusion for multiple mutations in the extended AGEZ of α-tropomyosin exon 3 [49,50]. Moreover, single nucleotide polymorphisms (SNPs) that affect the BPS have been shown to have a dramatic influence on the degree of exon inclusion or skipping [51]. Given the sensitivity of dBP exons to mutation within their AGEZ, it is interesting to note that many of the exons with AGEZ ≥ 150 are within genes that are either already known to be disease associated or are in some other way of biomedical interest. So far, we are not aware of any disease causing mutations within the AGEZs of dBP exons. However, there are a number of intronic SNPs within some of them (for example, two within the AGEZ of GABBR1 exon 23; Figure 5), and it is possible that some of these could modulate alternative splicing of their associated exons. Indeed, awareness of the possibility of dBPs, as suggested by the presence of a large AGEZ, might help to improve the design of diagnostic scans. For example, exons 3, 4 and 5 of the serotonin 5-HT_4 _receptor gene (*HTR4*) have AGEZs of 149, 291, and 221 nucleotides (IDB1090103.1894, IDB1090103.27415, and IDB1090103.40737), an arrangement that is conserved in the murine ortholog. Polymorphisms in *HTR4 *have been associated with bipolar disorder and schizophrenia [52,53]. However, the PCR primers used to detect polymorphisms were proximal to the exons and would have missed potentially interesting SNPs further upstream within the extended AGEZs.

An interesting feature of the regulation of dBP exons is that the small group that have been analyzed experimentally are all regulated by PTB [20,21,24,54,55]. It will be of interest to determine whether this is a general feature of dBP exons or is merely a coincidence, and also to investigate whether the dBP organization is associated with particular types of tissue specificity of regulation. The collection of extended AGEZs should also provide an enriched source of sequence elements involved in splicing regulation.

## Conclusion

We have characterized a group of human exons based upon the large size of the AGEZ immediately upstream. We have verified the location of the dBP toward the 5' end of some of these large AGEZs. Exons with large AGEZs have a higher incidence of computationally observed alternative splicing. If the common rationale for the dBP arrangement is to have regulatory elements located between the dBP and exon, then it is likely that many or most of the dBP exons will ultimately prove to be alternatively spliced, and that initial characterization of a large AGEZ may be a predictor of alternative splicing. These exons are also of interest because they would be vulnerable to mutations within their entire AGEZ that could lead to modification or even loss of gene function. We plan to develop our data set of dBP exons further with the aims of improving our predictions for the likely location of dBPs, and of improving the annotation of the database entries to include evidence of alternative splicing, locations of known SNPs, or mutations, and the consequences of these known sequence variants.

## Materials and methods

### Computational methods

#### Computational base data

The altExtron data set of transcript confirmed introns and exons was used as base data (altExtron version 3; based on GenBank version 147) [31,32,56]. This provides a cleaned data set of transcript confirmed introns and exons in a convenient flat-file format. From these data we extracted 67,334 human introns (excluding AT-AC introns), belonging to 10,527 distinct genes. Here we are considering acceptor (3') splice sites, and refer to the splice site, downstream exon, or upstream intron as best suits the context. For each intron/exon we also extracted from altExtron its status regarding observed alternative splicing, indicating whether the exon had been observed as absent in some transcripts (a cassette exon type alternative splicing event), and/or whether there was any observed alternative acceptor splice sites (leading to exon truncation or extension).

#### Definition of the AGEZ, the region of interest (ROI), and modified exclusion zone values

For each exon/intron under consideration, the AGEZ was defined as the distance from the acceptor splice site to the first upstream AG, ignoring any AG found in the first 12 nucleotides (as explained under Background, see above). Note that AG dinucleotides are usually absent from this region (in >90% of cases) compared with equivalent regions downstream of acceptor sites or on either side of randomly sampled AGs within the pre-mRNA sequences (for all of which absence of flanking AGs occurs at around 40%). We also scan further upstream for the second and third AG, and downstream for the first and second downstream AG dinucleotides. A ROI was defined as spanning from the third AG upstream to the second AG downstream. Note that the ROI extends to the third upstream AG for two reasons. First, AGs that are not used as 3' splice sites can be located within a short distance downstream of the BPS [28,30,45]. In addition, if the second and third AGs are also widely separated, then this may indicate that the large AGEZ is not associated with a dBP (see Results, above). We proceeded to build a flat-file that, for each intron/exon, included the sequence of the ROI (broken into the upstream and downstream components), the positions of the identified AG dinucleotides, and the positions of putative PPT and U2 BPS (described below). This flat-file forms the base data set for ongoing computational work into the sequence elements that define and constrain acceptor splice sites.

For the purposes of constructing and analyzing Figure 2, modified exclusion zone (mod-EZ) values were used. For each dinucleotide, we defined mod-EZ_1 _by searching upstream from position -25 (relative to the 3' splice site) for the first occurrence of the dinucleotide. A further shift of -25 from the AG that terminated the corresponding mod-EZ_1 _was performed before commencing the search to define the mod-EZ_2_. This definition acts to exclude the region immediately upstream of the 3' splice site within which the PPT is most often found, and hence minimize bias in the EZ_1 _distributions caused by this pyrimidine-rich region. Furthermore, we have observed that the occurrence of an AG (and all other dinucleotides) is not an independent event in that the observed probability that a dinucleotide under consideration is an AG is greater if there is a nearby AG than otherwise (data not shown). Thus, by including the -25 shift at the start of the mod-EZ_2 _search, we treat the EZ_1 _and EZ_2 _searches equally in this regard. Note that use of these mod-EZ values is expected to be conservative in demonstrating the postulated differences between the AGEZ_1 _and AGEZ_2 _distributions. Finally, and again just for the purpose of constructing Figure 2, introns of length less than 350 nucleotides were excluded for the following reasons: first, we observe overall an increased frequency of AG dinucleotides in exons compared to introns (by close to 10%; data not shown); and, secondly, the last two nucleotides of an exon are AG in around 50% of cases. Hence, we do not want the EZ_2 _to extend into exonic regions, which is avoided in all but the most extreme cases by application of this length restriction. Note that 50% of mod-AGEZ_1 _values are ≤ 13 nucleotides and 90% are ≥ 40 nucleotides.

#### Base composition issues, interpretation of the mod-AGEZ1 shoulder, and predicted frequency of distant branch points

Heterogeneity in the base composition between sequences is an important and often problematic aspect of analyses of the sort presented here. In addition to the consequences arising from the dynamics of overall compositional biases, there may also be subsets of the data in which aspects of the composition are under quite specific selective pressures; indeed, the behavior of AG dinucleotides that we are examine here is precisely such an effect and it might be that there are numerous other phenomena of this flavor affecting the composition of subsets of the data. As a first step to ameliorating the consequences of such effects, we designed the analysis around the comparison of EZ_1 _and EZ_2 _distributions, in which each intron contributes one datum to each of these distributions (for each dinucleotide under consideration).

Initially we attempted to model the curves as resulting from a process analogous to a series of coin tosses, in which the chance of observing the terminating dinucleotide at each step in the scan was a constant, like that of obtaining a head in the toss of a fair coin, which remains one half irrespective of what has happened previously. This assumption allowed us to model these EZ curves as negative binomial distributions and, we thought, this would lead to straightforward quantification of the differences between the pairs of EZ_1 _and EZ_2 _distributions. We were unable to obtain good and robust fits of this model to the data, which led to exploration of the underling base composition dynamics. Importantly, we found that (for all dinucleotides) the overall chance of terminating at the next step in the scan decreased substantially as the distance scanned increased (data not shown). We came to understand this effect as resulting from heterogeneity across the gene sequences; that is, as the region scanned becomes long it becomes more probable that the gene/intron being examined has base composition dynamics that tend to exclude the dinucleotide under consideration. Although these facts prevented our analysis from proceeding along the path of fitting distributions to the data, Figure 2 suggests that - at a gross level - these dynamics affect the EZ_1 _and EZ_2 _distributions equally.

We also specifically examined the overall observed probability of an AG occurring close to either a 3' splice site AG or an AG randomly selected from within the pre-mRNA sequence. A randomly selected AG was seen to have a 60% chance of having another AG in the 12 nucleotides immediately upstream, as compared with only around 10% for a 3' splice site AG. In both cases the region 12 nucleotides immediately downstream also had a 60% chance of containing an AG. Thus, the regions immediately upstream of 3' splice site have a greatly decreased occurrence of AG, leading to a concern that comparison of simple EZ distributions (in which the EZ_2 _starts immediately where the EZ_1 _ends) was not a fair comparison. That is, the EZ_2 _values would tend to be shorter than they might otherwise be because scanning starts in a region that has an increased chance of containing the dinucleotide under consideration. A further related concern is that, although heterogeneity between the sequences in the data set may be contained, heterogeneity within an intron could affect our analysis. The positional bias in pyrimidine composition within introns is an obvious candidate for introducing this sort of bias. It was for these reasons that the mod-EZ definition was developed, whereby the inequality between the AGEZ_1 _and AGEZ_2 _distributions arising from starting the AGEZ_2 _scan immediately where the AGEZ_1 _terminated was avoided, and whereby much of the bias that might arise from the presence of PPTs immediately upstream of 3' splice site was also avoided.

The analysis of Figure 2 given in the Results section (see above) focused on the shoulder observed for mod-AGEZ_1_, and specifically that there are 148 introns with mod-AGEZ_2 _≥ 100 and 279 such introns for the mod-AGEZ_1 _curve, leading to a χ^2 ^value of 116 and a *P *value close to zero. An equivalent analysis of the other dinucleotides was undertaken as follows: determine the position for the mod-EZ_2 _curve at which its tail contains close to but no fewer than 148 introns, this being in order to have analysis equivalent to the AG case; and, using this cutoff position, compare with the tail of the corresponding mod-EZ_1 _curve (Table 1). It was thus seen that significant differences also exist for other dinucleotides. In addition to the stand out case of AG, it was seen that highly significant biases exist toward TC and CT and against AA, GA, and GG in the mod-EZ_1 _regions; that there is substantially significant bias towards TA and CA; that there is marginally significant bias toward TG and against GC and AT; and that there is no significant bias for AC, CC, CG, GT and TT.

Examination of Table 1 revealed a series of biases that strongly suggest that the presence of PPT sequences in some mod-AGEZ_1 _regions were acting to generate for GA, GG and AA lesser biases of the same sort observed for AG. For instance, if our model had been that scanning took place for a GA, then we might conclude that a GAEZ_1 _of ≥100 nucleotides had an approximately 158/211 (75%) observed probability of arising by chance alone, and the complementary 25% observed probability of indicating a dBP. We have no reason to suppose there is any such scanning for GA, and every reason to suppose that this difference between the mod-GAEZ_1 _and mod-GAEZ_2 _curves is a straightforward consequence of PPT sequences in some mod-GAEZ_1 _regions acting to bias the mod-GAEZ_1 _distribution to higher values. Should we attribute, for AG, this same effect to some part of the 47% that we have provisionally attributed to dBPs? For the AA, GA, and GG binucleotides there are tail mod-EZ_1 _excesses as for AG of 33%, 25%, and 25%, respectively, averaging to 28%; if a correction were to be applied for AG, then it would discount the 47% by this amount to 19%.

A further simple analysis sheds some light on this question. We broke the mod-AGEZ_1 _distribution into two parts on the basis of there being an AG in the region -12 to -25 (the 'plus' group), and the complementary 'minus' group without an AG in this region. We then looked at the fraction of the mod-AGEZ_1 _values ≥100 nucleotides for each of these two groups and found for the plus group 22/10,330 (0.21%) of the mod-AGEZ1 values at ≥ 100. In contrast, if there were no AG in the region -12 to -25 (the minus group), then we see 257/39,546 (0.65%) with mod-AGEZ_1 _≥ 100. With a null hypothesis that the minus group should generate the same statistics as the plus group, we see the null hypothesis to be false with a χ^2 ^of 356 (*P* ≈ 0). This compares with tails ≥ 100 for the mod-AGEZ_1 _and mod-AGEZ_2 _curves shown in Figure 2 of 0.57% and 0.30%, respectively. That the magnitude of the mod-AGEZ_1 _tail is less than that for the 'minus' group above (at 0.58% compared to 0.65%) is expected because the mod-AGEZ_1 _includes both the plus and minus groups (and is thus a conservative measure). That the magnitude of the mod-AGEZ_2 _tail is greater than the 'plus' group (at 0.30% versus 0.21%) is not statistically significant (*P *= 0.2), and in any case is expected because the presence of an AG in -12 to -25 increases the chance of observing an AG shortly after -25 compared with the minus group. This analysis confirms that the shoulder seen for mod-AGEZ_1 _is not a general feature of the sequence composition at the 3' ends of introns independent of the splicing signals, but rather is a consequence of dBPs.

There are at least two ways to think about the difference between the mod-AGEZ_1 _and mod-AGEZ_2 _curves in Figure 2. On the one hand it may seem the curves demonstrate that if an AGEZ value of 100 nucleotides or more is observed, then there is an approximately 50% (148/279 = 0.53) probability that this has arisen by chance alone, and a complementary 50% probability that a dBP is the causative factor. On the other hand it may seem that the presence of PPTs at the 3' end of introns can push the mod-AGEZ_1 _distribution toward higher values and thus introduce a bias that is not fully accounted for (as above). If it is accepted that the scanning model is true, along with the implication that AG dinucleotides will only in rare circumstances be found in the region from about 15 nucleotides downstream of the BPS to about 12 nucleotides upstream of the 3' splice site, then the fact that PPTs influence the base composition in this region is a consequence of the position of the BPS, and thus is immaterial in relation to interpretation of the shoulder observed for mod-AGEZ_1 _in comparison with mod-AGEZ_2 _in Figure 2. This is what we contend. If our contention is not accepted, then it is necessary to discount the portion of AGEZ_1 _values ≥ 100, indicating a dBP from around 50% to around 20% (as above).

#### Identification of putative polypyrimidine tract and branch point sequence signals

Putative PPTs were identified in the ROI, as defined by Clark and Thanaraj [31]. Putative U2 BPS identification utilized the heptamer consensus YNYURAY (ideally UACUGAC) and the AGEZ according to the following heuristic procedure (following [31]): for introns with an AGEZ ≤ 40 nucleotides, a sequence fragment equal in length to the AGEZ but shifted upstream 15 nucleotides was searched for matches to the above consensus allowing a single mis-match at any position other that the branch point adenosine; in cases in which one and only one such match was found, this sequence contributed to the building of a weight matrix that was then used to search the ROI of all introns to identify and score putative U2 BPS. The derived weight matrix is given in Table 2 and may be contrasted with the weight matrix used in recent work from the Ast laboratory [45] that is given in Table 3. Although these two tables show some minor differences, it is difficult to draw any strong conclusions given the relatively small number (19) of sequences used by Kol and coworkers [45] to build Table 3. It is, however, noted that our method essentially reflects the consensus sequence used to build it.

### Output files

The flat files of acceptor splice sites, with information about putative PPT and U2 BPS is available online [33] and contains entries of the form:

>IDB1072296.1230

GB_MAP: IDB1072296 = A06939.1 (1..5322)

PROD: furin

AGEZ: 27

ROI: 1181..1265 -> -50..34

AG: -50, -47, -29, -2, 3, 35,

PPT: -42..-33, -23..-5, 5..14,

U2BP: -31 [5.49], -24 [5.2], 11 [4.58],

SEQ1: agaaggcaCTCTGTGCCTgacagctgaCCCTACCTTCCCTGTCCCCacag

SEQ2: tgagCCACTCATATggctacgggcttttggacgcag

END

'IDB1072296.1230', in this case, is the altExtron identifier for the gene (IDB1072296), with a transcript confirmed intron having a 3' splice site position (1230) being the position of the final intronic nucleotide. 'GB_MAP' gives the mapping to the GenBank entry from which this gene was derived as the accession, version, and (region). If the gene is on the complement strand in GenBank (always sense in altExtron), then the mapping will be labelled as complement. 'PROD' is the gene product as parsed from the GenBank flat files in the construction of altExtron. 'AGEZ' gives the AG exclusion zone. 'ROI' gives the ROI (see above), first in gene coordinates and then relative to the 3' splice site (with no position 0). 'AG' lists the relative positions of the AG nucleotides in the ROI, including the splice site itself at -2. 'PPT' gives the relative positions of putative PPTs in the ROI. 'U2BP' gives the relative positions of putative U2 BPS, with the bracketed number being the bit score from the weight matrix analysis. 'SEQ1' gives the intronic sequence part of the ROI with the putative PPTs in upper case (this may wrap over several lines). 'SEQ2' gives (as for SEQ1) the exonic sequence part of the ROI. Finally, 'END' is a tag helpful in file parsing that indicates the end of the record.

#### Frequency of alternative splicing versus AG dinucleotide exclusion zone

We examined the level of observed alternative splicing as a function of the AGEZ. Transcript confirmed introns/exons were seen to undergo alternative splicing when they were overlapped by other transcript confirmed introns/exons, and this information was derived from the altExtron flat files. We considered only those observed alternative splicing events that unambiguously fitted into one of two classes of alternative splicing: cassette exon usage (of an exon adjacent to the 3' splice site under consideration), and exon modification at the acceptor site (extension or truncation by use of competing 3' splice site). In both cases we excluded from consideration (for Figure 7) any acceptor site where another acceptor site was observed ≥ 40 nucleotides upstream. This prevents such an upstream 3' splice site defining the AGEZ in all but extreme cases, and acts to select the upstream AG in cases where an isoform pair share a BPS and PPT but differ in the use of two closely spaced AGs for the 3' splice site. Acceptor sites were grouped according to AGEZ value, initially into decade bins, with these then combined as necessary to ensure a minimum of 50 entries per group; as plotted in Figure 7, the fraction of each group with observed alternative splice isoforms was calculated. The standard error was calculated as sqrt(*r*·(*n *- *r*)/*n*), with *n *being the total number of introns in the group, and *r *being the number of these seen to undergo alternative splicing of the defined type.

For the purposes of statistically testing the hypothesis that exons with large AGEZ values are observed to be alternative exons at a higher frequency than exons with lesser AGEZ values, a cutoff of 100 nucleotides was used to define 'large'. Above this cutoff, 68 out of 235 exons were seen to be cassette exons, as compared with an expected value of 47 (on the basis of the overall average of 19.8%); this leads to a χ^2 ^value of 9.4, giving a *P *value of 0.002.

### Molecular and cell biology

#### Constructs

PAC clones, RPI-271M21 (IDB1089010) and RP5-1009E24 (IDB1088375), were obtained from The Sanger Institute Clone Resources Group. PAC clones were tested for bacteriophage contamination by standard procedures [57]. The ROI was PCR amplified using *Pfu *polymerase, treated with *Taq *polymerase to add an A overhang, and ligated into pGEM-Teasy (Promega, Madison, WI, USA) as a shuttle vector.

PCR primers for IDB1089010 were as follows: forward 5'-CCTCTAGTAGTCAACACTCACAGCAGC; and reverse 5'-GGATAGCATGTTCTTCCCAGCTGG. PCR primers for IDB1088375 were as follows: forward 5'-CCCAAAGTGTTGGGATTACAGG; and reverse 5'-CGGACGAATTCTGTCTGCGTTGAC.

Branchpoint and upstream AG mutations were carried out using QuikChange Site-Directed Mutagenesis (Stratagene, La Jolla, CA, USA) in the Teasy clones. Wild-type and mutant clones were subcloned using standard cloning techniques [58] from the shuttle vector either as a *Not*I fragment into pCAGGsEGFP [21,59] or as an *Eco*RI fragment into pTS3St [26,60]. All subcloned fragments were sequenced in their entirety to ensure that no constructs had secondary mutations.

#### Cell culture, transfection, and analysis of cellular RNA

HeLa cells were grown in Dulbecco's modified Eagles medium containing 10% foetal calf serum. Transient transfection was carried out using Lipofectamine (Invitrogen, Carlsbad, CA, USA), total RNA was isolated using TRI reagent (Sigma, Poole, Dorset, USA), and RT-PCR was carried out as previously described [55]. Primers for pCAGGsEGFP based constructs were as follows: RT primer 3'CGRT, 5'-TAGTTGTACTCCAGCTT; forward 5'CGTM, 5'-GGCAAAGAATTCGCCACCA; and reverse 3'CGTM, 5'-GGGTGTCGCCCTCGAACTT. Conditions for the PCR were 30 cycles of 94°C at 30 s, an annealing temperature of 58°C for 30 s, followed by an extension at 72°C for 1 minute using a MgCl_2 _concentration of 1.5 mmol/l.

Primers for pTS3St based constructs were as follows: RT primer SV3'RT, 5'-GCAAACTCAGCCACAGGT; forward SV5'2, 5'-GGAGGCCTAGGCTTTTGCAAAAAG; reverse SV3'1, 5'-ACTCACTGCGTTCCAGGCAATGCT. Conditions for the PCR were 30 cycles of 94°C for 30 s, an annealing temperature of 62°C for 30 s, followed by an extension at 72°C for 1 minute using a MgCl_2 _concentration of 2.5 mmol/l.

#### *In vitro *splicing

*In vitro *transcription and splicing were carried out as previously described [55,60].

## Additional data files

The following additional data are included with the online version of this article: A data file containing the complete set of sequences used in the analysis (Additional data file 1) and a data file with the subset of entries with AGEZ> = 150 nt (Additional data file 2) Data are also available online [33]. Scripts are available on request.

## Supplementary Material

Additional data file 1 The complete set of sequences used in the analysis Click here for file

Additional data file 2 A data file with the subset of entries with AGEZ> = 150 nt Click here for file

## Figures and Tables

**Table 1:** Analysis of the curve pairs from Figure 2

Dinucleotide	C	EZ_2_	EZ_1_	χ^2^
AA	218	148	211	26.8
AC	143	149	129	2.7
AG	100	148	279	116.0
AT	186	149	173	3.9
CA	98	151	187	8.6
CC	227	151	161	0.7
CG	1,039	148	150	0.0
CT	96	150	86	27.3
GA	120	158	211	17.8
GC	186	148	173	4.2
GG	203	150	200	16.7
GT	111	149	153	0.1
TA	292	149	185	8.7
TC	131	150	77	35.5
TG	74	149	123	4.5
TT	177	148	157	0.5

Shown is an analysis of the curve pairs from Figure 2 comparing the tails of the modified exclusion zone (mod-EZ)_1 _and mod-EZ_2 _distributions for each dinucleotide above a cutoff (C). This cutoff is the point at which the mod-EZ_2 _tail contains close to but no fewer than 148 entries (in order to have analysis equivalent to the AG case). The EZ_2 _and EZ_1 _columns give the observed numbers of entries above C, and the χ^2 ^column gives the associated χ^2 ^value. Note that, with one degree of freedom, a χ^2 ^value of 4 gives a *P *value close to 0.05; thus, χ^2 ^values < 4 are not statistically significant.

**Table 2:** Derived weight matrix for identifying and scoring human branch point sequences

A	0.090	0.247	0.037	0.090	0.359	1.000	0.071
C	0.430	0.283	0.583	0.048	0.087	0.000	0.469
G	0.084	0.216	0.070	0.048	0.517	0.000	0.127
T	0.395	0.254	0.311	0.814	0.036	0.000	0.333

**Table 3:** Weight matrix from 19 experimentally determined human branch point sequences

A	0.158	0.368	0.000	0.000	0.263	1.000	0.211
C	0.368	0.211	0.632	0.211	0.316	0.000	0.526
G	0.211	0.158	0.158	0.000	0.263	0.000	0.053
T	0.263	0.263	0.211	0.789	0.158	0.000	0.211

The experimentally determined human branch point sequences were reported by Kol and coworkers [45].
